# A Web-Based Intervention (Germ Defence) to Increase Handwashing During a Pandemic: Process Evaluations of a Randomized Controlled Trial and Public Dissemination

**DOI:** 10.2196/26104

**Published:** 2021-10-05

**Authors:** Sascha Miller, Ben Ainsworth, Mark Weal, Peter Smith, Paul Little, Lucy Yardley, Leanne Morrison

**Affiliations:** 1 Center for Clinical and Community Applications of Health Psychology Department of Psychology University of Southampton Southampton United Kingdom; 2 Bath Centre for Mindfulness and Compassion Department of Psychology University of Bath Bath United Kingdom; 3 Web and Internet Science Group Electronics and Computer Science University of Southampton Southampton United Kingdom; 4 Department of Social Statistics and Demography School of Economic, Social and Political Scientces University of Southampton Southampton United Kingdom; 5 Primary Care and Population Sciences School of Medicine University of Southampton Southampton United Kingdom; 6 Centre for Academic Primary Care School of Psychological Science University of Bristol Bristol United Kingdom

**Keywords:** behavior, infection, prevention, respiratory tract infection, internet, evaluation studies, pandemic, COVID-19, transmission, virus, influenza, respiratory, intervention, digital intervention, dissemination

## Abstract

**Background:**

Washing hands helps prevent transmission of seasonal and pandemic respiratory viruses. In a randomized controlled trial (RCT) during the swine flu outbreak, participants with access to a fully automated, digital intervention promoting handwashing reported washing their hands more often and experienced fewer respiratory tract infections than those without access to the intervention. Based on these findings, the intervention was adapted, renamed as “Germ Defence,” and a study was designed to assess the preliminary dissemination of the intervention to the general public to help prevent the spread of seasonal colds and flu.

**Objective:**

This study compares the process evaluations of the RCT and Germ Defence dissemination to examine (1) how web-based research enrollment procedures affected those who used the intervention, (2) intervention usage in the 2 contexts, and (3) whether increased intentions to wash hands are replicated once disseminated.

**Methods:**

The RCT ran between 2010 and 2012 recruiting participants offline from general practices, with restricted access to the intervention (N=9155). Germ Defence was disseminated as an open access website for use by the general public from 2016 to 2019 (N=624). The process evaluation plan was developed using Medical Research Council guidance and the framework for Analyzing and Measuring Usage and Engagement Data. Both interventions contained a goal-setting section where users self-reported current and intended handwashing behavior across 7 situations.

**Results:**

During web-based enrolment, 54.3% (17,511/32,250) of the RCT participants dropped out of the study compared to 36.5% (358/982) of Germ Defence users. Having reached the start of the intervention, 93.8% (8586/9155) of RCT users completed the core section, whereas 65.1% (406/624) of Germ Defence users reached the same point. Users across both studies selected to increase their handwashing in 5 out of 7 situations, including before eating snacks (RCT mean difference 1.040, 95% CI 1.016-1.063; Germ Defence mean difference 0.949, 95% CI 0.766-1.132) and after blowing their nose, sneezing, or coughing (RCT mean difference 0.995, 95% CI 0.972-1.019; Germ Defence mean difference 0.842, 95% CI 0.675-1.008).

**Conclusions:**

By comparing the preliminary dissemination of Germ Defence to the RCT, we were able to examine the potential effects of the research procedures on uptake and attrition such as the sizeable dropout during the RCT enrolment procedure that may have led to a more motivated sample. The Germ Defence study highlighted the points of attrition within the intervention. Despite sample bias in the trial context, the intervention replicated increases in intentions to handwash when used “in the wild.” This preliminary dissemination study informed the adaptation of the intervention for the COVID-19 health emergency, and it has now been disseminated globally.

**Trial Registration:**

ISRCTN Registry ISRCTN75058295; https://www.isrctn.com/ISRCTN75058295

## Introduction

Pandemic respiratory viruses present a global health threat, leading to more deaths across a wider spread of the population than seasonal flu, as seen through outbreaks such as SARS-CoV (severe acute respiratory syndrome), swine flu (H1N1 influenza), MERS-CoV (Middle East Respiratory Syndrome), as well as COVID-19 (SARS-CoV-2) [1]. However, for more vulnerable groups such as the older adults or seriously ill, seasonal flu can still present a serious health risk and increased likelihood of needing medical care [1]. Handwashing is an accessible and a simple infection control behavior [2,3] that has been promoted to the general public for many decades to slow the spread of cold and flu viruses both seasonally and during pandemics [4,5]. The COVID-19 pandemic has seen renewed calls for increased handwashing from governments and health organizations around the world as a means to control the spread of the virus [6,7]. Yet, despite high levels of health promotion and public awareness of handwashing, evidence from prior pandemics (ie, SARS-CoV and swine flu) suggests that increases in the reported levels of handwashing were low [8,9]. An effective, evidence-based behavioral intervention to support increased handwashing within the home in the event of pandemics such as COVID-19 and for seasonal respiratory tract infections is urgently required [10-13]. Digital interventions provide the advantages of quick dissemination and flexibility so that contents can be updated to reflect the changes during a pandemic.

PRIMIT (PRImary care trial of a website based infection control intervention to Modify Influenza-like illness and respiratory infection Transmission) was commissioned and funded by the UK Medical Research Council in 2008 for use in the event of a pandemic [14]. A theory-based, stand-alone, digital intervention to increase handwashing targeting the general population was developed using the person-based approach [15]. This included extensive mixed-methods evaluation of the usability, functionality, and acceptability of the intervention [3,16,17]. A randomized controlled trial (RCT) of over 20,000 participants was carried out in the United Kingdom while swine flu was circulating in the community. The findings of this RCT established that participants who had access to the intervention reported washing their hands more than those who did not: they and the people they lived with had lesser respiratory tract infections and users who contracted respiratory tract infections were ill for lesser time [14]. A behavioral analysis of the data collected during the RCT demonstrated that viewing each of the 4 available sessions led to additive increases in handwashing levels [18]. However, completing the first session was deemed to be effective engagement; after viewing this session, the majority of users increased their handwashing to sufficient levels to lower the transmission of viruses [18,19]. This session contained a range of motivational messages and included a goal-setting behavior change technique (BCT). The goal-setting section required users to consider their current handwashing frequency across a range of specific situations (eg, after going to the toilet, before eating a meal) and then make a plan to increase handwashing in the future. Based on the findings from the behavioral analysis [18], in 2016, the architecture of the intervention was adapted to enable access to all the intervention contents in 1 session (rather than 4 sequential sessions). The intervention was renamed as “Germ Defence” and disseminated to the general public to lower the transmission of seasonal cold and flu viruses [20]. The data generated by the PRIMIT and Germ Defence studies provide us with the opportunity to compare the uptake and usage of the intervention in the RCT and “in the wild” contexts.

RCTs are considered to be the gold standard for evaluating intervention efficacy. However, they have been criticized for producing results that are not replicated once an intervention is freely disseminated to the intended population [21-23]. RCTs require artificial conditions that differ from how an intervention may be used “in the wild.” For example, RCTs of digital interventions typically involve effortful web-based research procedures such as lengthy enrollment processes, followed by in-depth baseline, interim, and follow-up data collection across several weeks, if not months [24]. These demanding procedures may affect both the type of people who take part in an RCT and how they use the intervention [21,25,26]. Participants with lower levels of education or health literacy are more likely to drop out of a study during these processes [27], meaning that these groups are unintentionally excluded. Conversely, this can lead to volunteer bias within the sample [26], whereby people who are more highly motivated to perform the behavior or have higher levels of health literacy/education are more likely to participate in the trial. In addition, research suggests that effortful procedures may increase participants’ sense of support or accountability, leading to artificially high levels of engagement [21].

Calls have been made to evaluate digital interventions so that their effectiveness may be established beyond trial conditions [24]. However, a review of studies of publicly available digital interventions found that few reported in-depth usage data and only 1 intervention was identified as having been empirically examined through an RCT as well [21]. Having established the effectiveness of the PRIMIT intervention in a trial context, the Germ Defence study was subsequently devised as a novel preliminary dissemination of the intervention to the general public. Comparing intervention usage across these 2 contexts provides the opportunity to examine the impact of the rigors of RCTs on intervention usage and effectiveness and to assess whether the intervention maintains efficacy when accessed “in the wild” [28,29].

This paper reports process evaluations [30] of the PRIMIT and Germ Defence studies. The aim of this study was to provide insights that would help to optimize the intervention and maximize reach in the event of a pandemic and to provide a comparison between RCT and “in the wild” contexts. This has proved timely, as the Germ Defence intervention has now been adapted and widely disseminated for use during the COVID-19 pandemic [31,32]. By comparing trial and “in the wild” contexts, we were able to examine the effect of the RCT research procedures on uptake and usage. Examining attrition during trial procedures has the potential to inform RCT design and produce findings that will translate when implemented at scale within the community. Further, examining the usage of intervention content across the 2 contexts provides insight into how to design interventions that are likely to be more effective and engaging when disseminated at scale. As the intervention is effective where other public health campaigns have had less success [8,9], it is important to understand how increased handwashing was achieved. The Germ Defence study enabled us to examine whether the goal-setting section continued to support increased intentions for handwashing in specific situations when the intervention is not supported by the trial context. The specific objectives of this study were to compare (1) completion of the web-based research enrollment procedures, (2) usage of the intervention content, and (3) self-reported intentions to wash hands in specific situations.

## Methods

### Study Procedures

**Figure 1:**
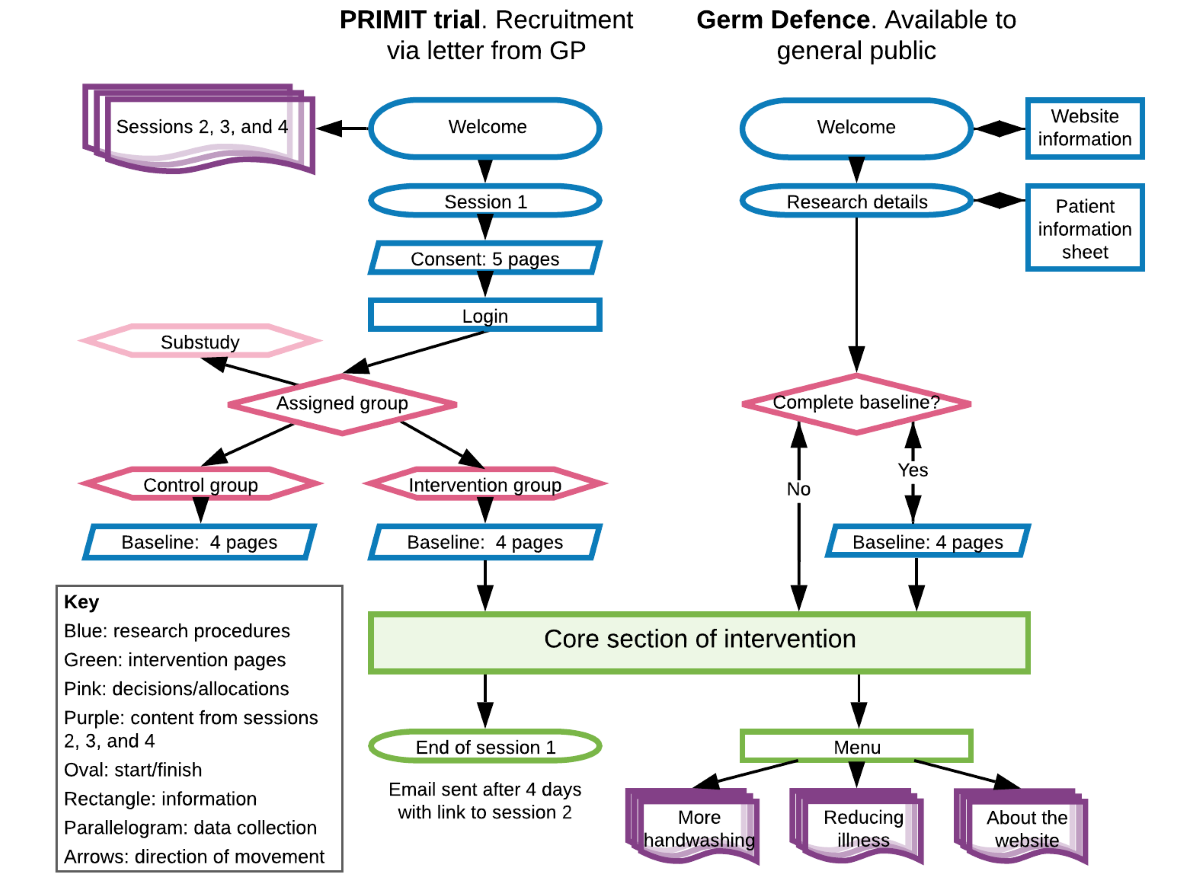
Research procedures for PRIMIT (PRImary care trial of a website based infection control intervention to Modify Influenza-like illness and respiratory infection Transmission) and Germ Defence studies.

The PRIMIT trial ran across 3 winters from 2010 to 2012. Participants were randomized equally to either the intervention or control groups. Both groups were required to give consent, log in, and complete baseline measures to enroll in the study. Participants in the intervention group were given immediate access to the first session of the website (Figure 1). This trial is registered with the ISRCTN registry number ISRCTN75058295; for full details of the trial, see [14].

Germ Defence was available from 2016 to 2019. On the webpages prior to accessing the intervention, users were provided with details of the study and asked if they would like to participate by providing baseline measures or go directly to the intervention. For users volunteering to complete baseline measures, the consent process was adapted to provide minimal but sufficient information for informed consent, with full details available as an optional click-through.

### Study Design

A process evaluation plan to compare usage of PRIMIT and Germ Defence studies and interventions was developed while adapting and preparing Germ Defence for dissemination in 2016. The plan was structured using the Analyzing and Measuring Usage and Engagement Data (AMUsED) framework [20,33-35] (see Multimedia Appendices 1-3) and is in line with Medical Research Council process evaluation guidance [30]. The process evaluation team combined expertise in psychology, primary care, statistical analyses, and computer science. Most team members were already familiar with the intervention having worked on the PRIMIT study development [3,16,17] or the RCT [14,18].

### Participants

PRIMIT study participants were recruited to the RCT by letters sent from National Health Service general practices to patients aged 18 years or older living with at least one other person who was willing to report their illnesses for the study and had internet access. Patients with severe mental problems, who were terminally ill, or who had a skin complaint that restricts handwashing were excluded from this study. Access to the website was restricted so that only users who received specific login details were able to enroll and consent to the study online. Participants were not compensated for taking part in this study.

The Germ Defence study was accessible to members of the general public who indicated being aged 16 years or older. Website details were distributed to various health sector organizations for use with their clients (eg, health support charities for people at risk from respiratory tract infections and local council public health organizations). Germ Defence is endorsed by the National Institute for Health and Care Excellence antimicrobial resistance guidelines and a link to the intervention is provided on their website. The intervention was also promoted directly to the general public (eg, through a chain of high-street pharmacies, play groups, social media, student intranet).

### Ethical Approval

The PRIMIT study was approved by a multicenter research ethics committee (08/H0502/14). The Germ Defence study was approved by the University of Southampton, School of Psychology ethics committee (19399).

### Measures

The PRIMIT study and Germ Defence websites were developed using LifeGuide software (University of Southampton) [36]. The software collected self-report measures and automatically recorded user interaction with the intervention such as time, date, pages viewed, and the order they were viewed in. For the Germ Defence study, web-based, self-reported baseline measures of user characteristics and behaviors were streamlined in comparison to those used in the PRIMIT study (see Table 1). Measures were selected based on the findings from the PRIMIT study [14] and completion of the AMUsED framework checklists [20]. By making the baseline measures voluntary, it was possible to split the sample into 2 groups: those who chose to complete these measures and those who did not.

**Table 1:** Germ Defence and equivalent PRIMIT web-based study measures.

Measures, items		Description	Response options	PRIMIT^a^ study	Germ Defence
**Baseline web-based self-report questionnaires**					
	Gender	Please select one	Male, Female, Other	✓	✓
	Age	Select age from dropdown box.	PRIMIT study: 18-99 years, Germ Defence: 16-99 years	✓	✓
	Recruitment	How did you hear about the website?	health support group, school or children’s group, search engine, social media, news article, word of mouth, other.		✓
	Current daily handwashing	How many times a day do you wash your hands, including using antibacterial hand gel, on average?	0-2, 3-4, 5-6, 7-9, 10 times or more.	✓	✓
	Intended daily handwashing	How many times a day do you intend to wash your hands in the future, including using antibacterial hand gel, on average?	0-2, 3-4, 5-6, 7-9, 10 times or more.	✓	✓
**Perceived risk of catching a respiratory tract infection**					
	Perceived likelihood of users catching a respiratory tract infection	PRIMIT: 2 items (eg, My chances of catching a cold or flu are high if I don’t take action to prevent it).	1-7 Likert scale ranging from “strongly disagree” to “strongly agree”	✓	✓
		Germ Defence: My chances of catching a cold, flu, or stomach bug are high if I don’t take action to prevent it.	1-7 Likert scale ranging from “strongly disagree” to “strongly agree.”		
	Perceived severity of catching a respiratory tract infection	If I catch a cold, flu, or stomach bug, I am likely to become seriously ill.	1-7 Likert scale ranging from “strongly disagree” to “strongly agree.”		✓
**Goal-setting section within the intervention**					
	Current behavior: Over the last week, I washed my hands	C1: Before I ate a meal	1=almost never, 2=sometimes, 3=quite often, 4=very often, 5=almost always	✓	✓
		C2: Before I ate snacks	1=almost never, 2=sometimes, 3=quite often, 4=very often, 5=almost always	✓	✓
		C3: After I went to the toilet	1=almost never, 2=sometimes, 3=quite often, 4=very often, 5=almost always	✓	✓
		C4: When I came in to the house	1=almost never, 2=sometimes, 3=quite often, 4=very often, 5=almost always	✓	✓
		C5: After I had been close to someone with a cold, flu, or upset stomach	1=almost never, 2=sometimes, 3=quite often, 4=very often, 5=almost always Germ Defence only: 0=not applicable	✓	✓
		C6: After blowing my nose or sneezing/coughing on my hands	1=almost never, 2=sometimes, 3=quite often, 4=very often, 5=almost always Germ Defence only: 0=not applicable	✓	✓
		C7: After touching something with lots of germs on	Germ Defence only: 1=almost never, 2=sometimes, 3=quite often, 4=very often, 5=almost always 0=not applicable		✓
	Intended behavior: I will try to wash my hands	Items as for current behavior	Responses as for current behavior		

^a^PRIMIT: PRImary care trial of a website based infection control intervention to Modify Influenza-like illness and respiratory infection Transmission.

### Intervention Structure

The intervention used for the PRIMIT and Germ Defence studies was developed by researchers at the University of Southampton. Full details of intervention structure and development are reported elsewhere [3,14,16,17] and archived copies are available [37] (see PRIMIT and Germ Defence v1). The intervention content for both the studies was “frozen” during the trial and dissemination periods.

**Figure 2:**
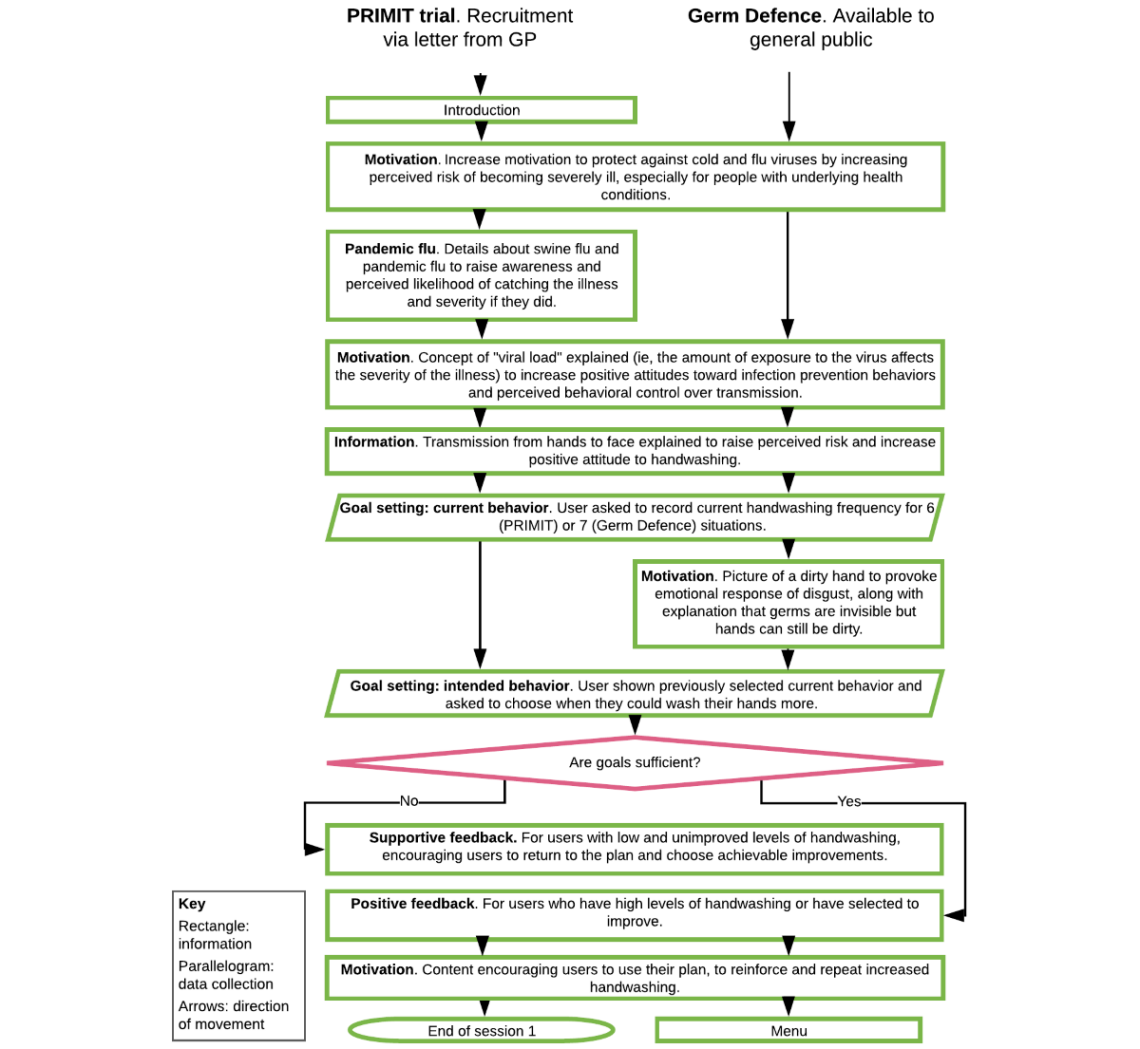
PRIMIT (PRImary care trial of a website based infection control intervention to Modify Influenza-like illness and respiratory infection Transmission) study and Germ Defence intervention structure for core section.

PRIMIT study users had access to 4 sessions of theory-based and evidence-based content released sequentially over 3.5 weeks. Having established that viewing the first session of the PRIMIT study intervention represented effective engagement [18], Germ Defence was also structured with the same content as the core section (Figure 2). After completing the core section, all information from the following 3 sessions was then available, allowing users to access the whole intervention in 1 visit, with no further sessions necessary. These core pages focused on informing, supporting, and motivating users to wash their hands more [3,17] and were “tunneled” so that users had to move through pages in a specific order. The appearance was updated for Germ Defence, but only 2 content changes were made to the core section: details about pandemic flu [17] and particularly the swine flu outbreak that started in 2009 were removed and a motivational message from the original “session 2” was added in between the goal-setting pages.

Within this core section was a goal-setting BCT encouraging handwashing in situations where there is increased risk of infection through spreading the virus inside the home (eg, when I come into the house) or transmission of viruses from hands to face (eg, before I eat snacks). These are the only pages in the intervention that require user interaction, and users were only able to progress further if they selected current and intended future handwashing for all situations. Users were provided with feedback after completing their plan: positive for high or improved levels of handwashing; supportive for low and unimproved levels of handwashing, suggesting users’ revisit and change of their plan. Having finished these pages, PRIMIT study users had completed session 1. For Germ Defence users, after this core section was a menu allowing users to access 3 further components with information from sessions 2-4 of the PRIMIT study. These included further support for handwashing and information on other infection prevention behaviors (ie, social distancing, not touching your face, wearing a mask, cleaning surfaces).

### Data Analysis

Anonymous usage data collected automatically through interaction with the intervention was examined for all Germ Defence users and PRIMIT participants in the intervention group. Usage of the core section of Germ Defence is reported for the whole sample and the subsample of users who completed baseline measures. Responses to self-report measures (ie, at baseline and goal-setting) were analyzed for the PRIMIT study intervention group and Germ Defence baseline respondents. As part of the PRIMIT trial requirement, a small proportion of users was placed into a substudy and they completed different baseline measures that excluded handwashing behaviors. As this group cannot be included in analyses with other users, they have been removed from this process evaluation.

SPSS for Windows version 25 (IBM Corp) was used for all statistical analyses. The frequency distribution of the scores for self-report measures was visually assessed for normality, and all measures of user characteristics at baseline were nonnormally distributed. All analyses were two-tailed. Owing to the difference in sample sizes, analyses of usage data are reported as percentages to enable more meaningful comparisons.

### Completion of Web-Based Research Enrollment Procedures

To examine the completion of the web-based research enrollment procedures, usage data were analyzed for the proportions of users who had viewed each page, continued on to another page, and left the study. Owing to changes in the data collection across the 3 winters, usage data covering research enrollment for the first 2 winters are not available (intervention usage data were unaffected). Therefore reported usage percentages for enrollment pages are taken from users recruited in the third winter, representing over 75% of the total sample. The number of views for the welcome pages (PRIMIT study n=209,852, Germ Defence n=12,106) included multiple views by the same participants, people who arrived at the page unintentionally, and bots (automated software programs). In addition, PRIMIT study participants who were returning to the intervention to view later sessions are included in this number. To enable equivalent comparisons between the 2 research procedures and to ensure that usage was intentional, the first page has been separated and proportions of participants have been calculated based on the sample who reached the second page.

### Intervention Usage

Intervention content usage was examined for the pages viewed and attrition in the core section of the intervention. Confidence intervals at 95% were used to compare the means of baseline behavioral and psychological measures to determine whether there were any differences between the intervention users in the two contexts. Logistic regression analyses were carried out to examine whether baseline user characteristics predicted completion of the core section of the intervention.

### Handwashing Behaviors

Confidence intervals at 95% were also used to compare the scores for intentions to handwash in the goal-setting situations. The first completion of intended handwashing responses for the goal-setting items was analyzed. Confidence intervals comparing individual handwashing situations are reported for practical significance, with mean differences >0.3 on a scale of 1-5. Paired sample cases items were excluded listwise to reduce any bias due to attrition, and equal variances were assumed for independent samples.

## Results

### Completion of Web-Based Research Enrollment Procedures

**Figure 3:**
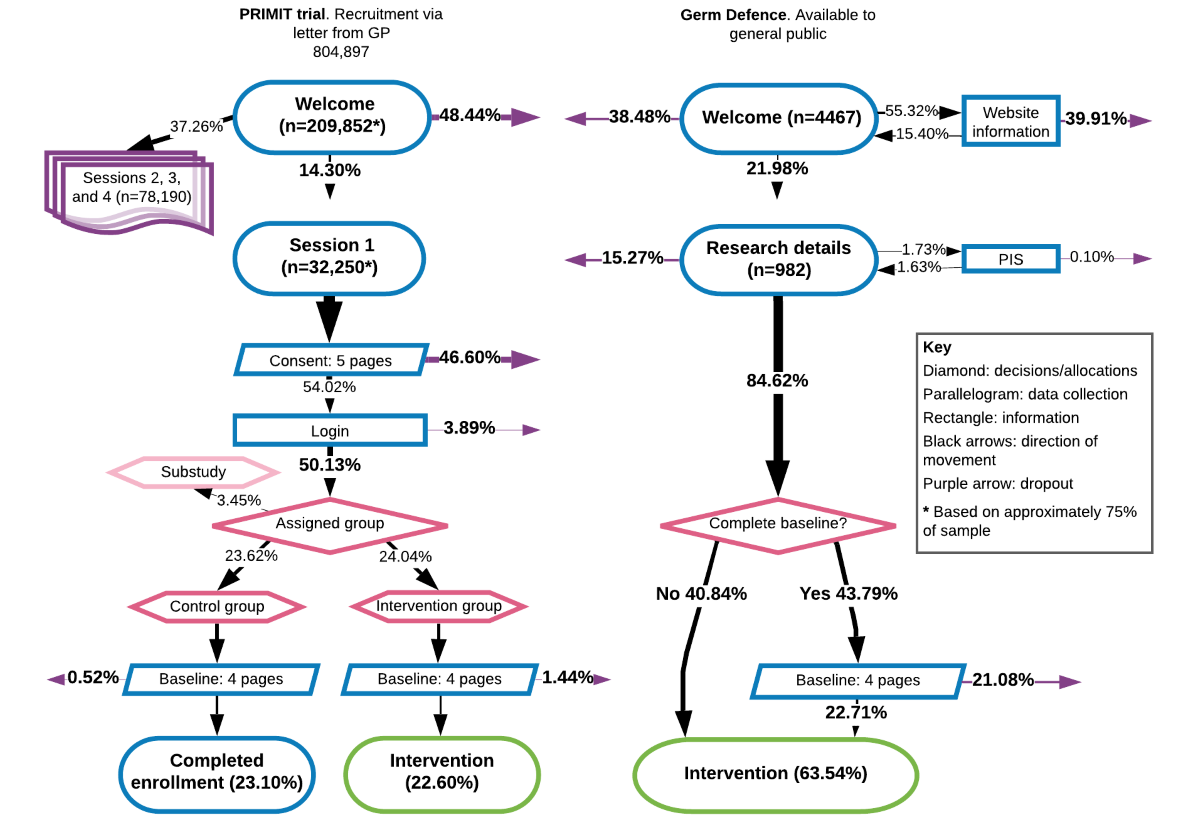
Flowcharts of usage during PRIMIT (PRImary care trial of a website based infection control intervention to Modify Influenza-like illness and respiratory infection Transmission) study and Germ Defence research procedures. Owing to the difference in sample sizes, analyses of usage data are reported as percentages to enable more meaningful comparisons. GP: general practice; PIS: patient information sheet.

Letters inviting patients to take part in the PRIMIT study were sent to 804,897 individuals, of which 2.5% (20,042/804,897) provided web-based consent, completed baseline measures, and were assigned to a group (Figure 3). The Germ Defence website received 12,106 visits to the first page. The majority of these were identified as bots (7639/12,106, 63.1%). Of the remaining 4467 visits, 4.9% (223/4467) continued to provide baseline measures and access the intervention and 8.9% (401/4467) accessed the intervention directly.

### Intervention Usage

#### User Characteristics

The sample population in Germ Defence study was younger (mean 42 years) and predominantly female (184/243, 75.7%) compared to that in PRIMIT (mean 57 years, 5002/8943, 55.9%). The samples were similar in most of the self-reported behaviors and beliefs at baseline (Table 2). PRIMIT study users reported higher intended levels of daily handwashing at baseline (mean 3.97) compared to current daily levels (mean 3.85; mean difference 0.118, 95% CI 0.107-0.130), as did Germ Defence users (intention mean 4.11, current mean 3.87; mean difference 0.239, 95% CI 0.131-0.348). Comparisons of scores between PRIMIT study and Germ Defence users for current (mean difference –0.022, 95% CI –0.172 to 0.127) and intended (mean difference –0.150, 95% CI –0.296 to –0.004) daily handwashing showed no practical significance. Germ Defence users perceived themselves as more likely to contract a respiratory tract infection (mean 5.53) than users in the PRIMIT study intervention group (mean 5.11; mean difference 0.418, 95% CI 0.199-0.637). None of the user characteristics recorded at baseline (eg, age, daily handwashing behavior) predicted completion of the core section for either PRIMIT or Germ Defence (Table S1 in Multimedia Appendix 4).

When asked how they had heard about the Germ Defence website, 236 users responded the following sources: news (41/236, 17.4%), word of mouth (21/236, 8.9%), social media (16/236, 6.8%), search engine (15/236, 6.4%), school or children’s group (14/236, 5.9%), and health support group (4/236, 1.7%); 125 (52.97%) users selected “other.” When asked to provide further details, 67 (28.4%) users identified themselves as working in health care–related professions. Owing to health care professionals’ (HCPs) familiarity with infection prevention behaviors, the characteristics of the identified HCPs were compared with those of non-HCPs within the Germ Defence study to examine any potential sample bias. When the sample was split by profession, HCPs indicated higher daily levels of current handwashing (mean 4.26) than non-HCPs (mean 3.71; mean difference 0.543, 95% CI 0.237-0.849). HCPs also selected higher daily intentions to wash their hands in the future (mean 4.51) compared to non-HCPs (mean 3.96; mean difference 0.550, 95% CI 0.243-0.856). Although HCPs perceived themselves as more likely to contract a respiratory tract infection (mean 6.00) than non-HCPs (mean 5.33; mean difference 0.667, 95% CI 0.213-1.121), the perceived likelihood of becoming very ill was similar across all Germ Defence baseline responders (HCPs mean 3.25; non-HCPs mean 3.62; mean difference 0.038, 95% CI –0.472 to 0.549).

**Table 2:** Characteristics of PRIMIT study intervention group and Germ Defence study baseline responders.

Measure		Participants (n)	Mean (SD)	Min-Max
**Responders**				
	PRIMIT^a^ study	8959	N/A^b^	N/A
	Germ Defence	250	N/A	N/A
**Gender split (female)**				
	PRIMIT study (n=8943)	5002	N/A	N/A
	Germ Defence (n=243)	184	N/A	N/A
**Age (years)**				
	PRIMIT study	8945	56.64 (13.631)	18-94
	Germ Defence	250	42.11 (13.035)	16-74
**Current daily handwashing**				
	PRIMIT study	8945	3.85 (1.150)	1-5
	Germ Defence	234	3.87 (1.094)	1-5
**Intended daily handwashing**				
	PRIMIT study	8944	3.97 (1.117)	1-5
	Germ Defence	231	4.11 (1.090)	1-5
**Perceived likelihood of user becoming ill**				
	PRIMIT study	8837	5.11 (1.652)	1-7
	Germ Defence	223	5.52 (1.582)	1-7
**Perceived severity for user**				
	PRIMIT study	N/A	N/A	N/A
	Germ Defence	223	3.61 (1.747)	1-7

^a^PRIMIT: PRImary care trial of a website based infection control intervention to Modify Influenza-like illness and respiratory infection Transmission.

^b^N/A: not applicable.

#### Intervention Content Usage

**Figure 4:**
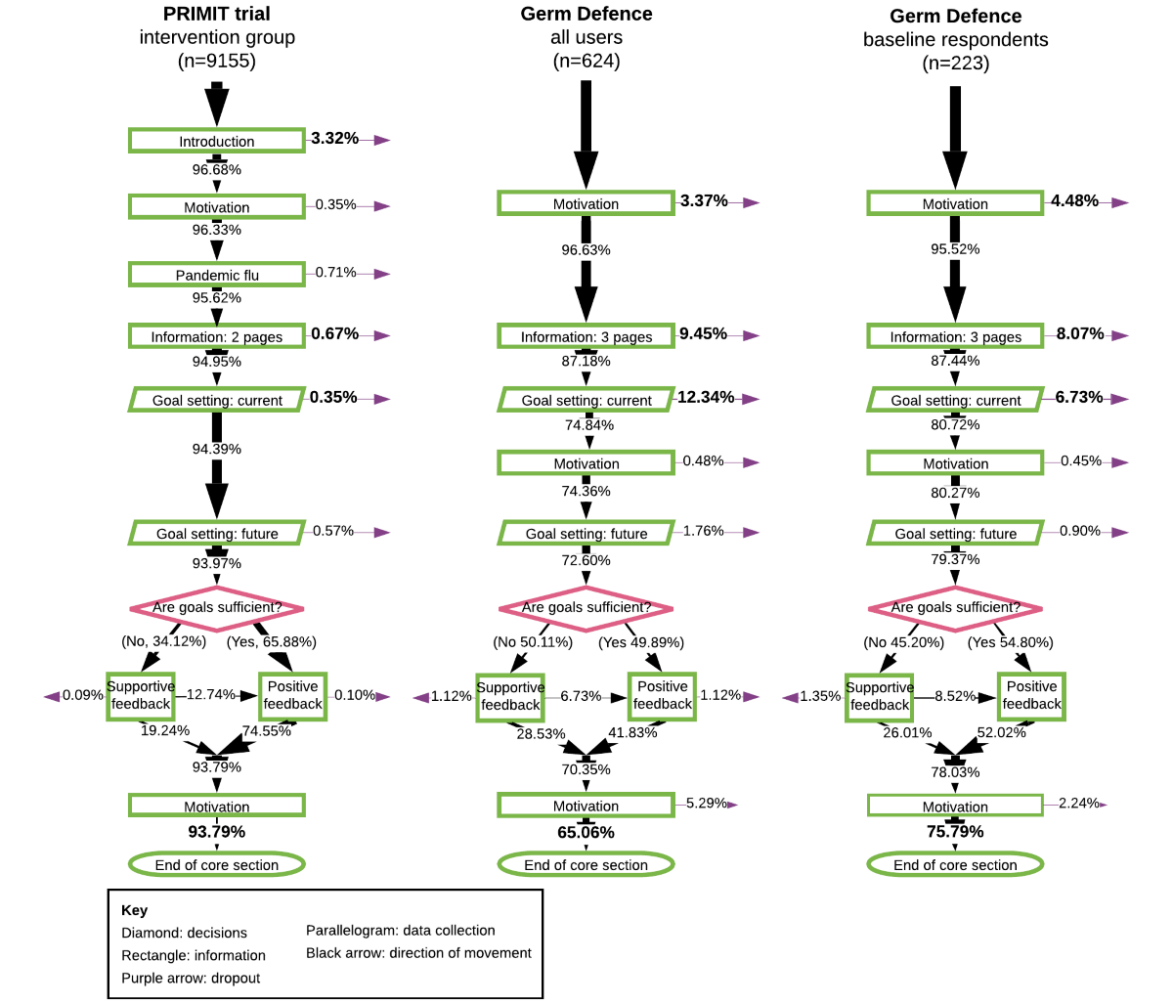
Flowcharts of percentage of usage for the core sections of PRIMIT (PRImary care trial of a website based infection control intervention to Modify Influenza-like illness and respiratory infection Transmission) and Germ Defence. Owing to the difference in sample sizes, analyses of usage data are reported as percentages to enable more meaningful comparisons.

Attrition was lower in PRIMIT, with fewer users failing to complete the core section (568/9155, 6.2%) compared to Germ Defence users (218/624, 34.9%) (see Figure 4). The pages that saw the most attrition in Germ Defence were those leading up to and including the first page of the goal-setting section, which saw a total of 25.2% (157/624) of users leave the intervention, accounting for 72.0% (157/218) of all attrition. Germ Defence users who had volunteered to complete baseline measures had lower levels of attrition across all of the core section (54/223, 24.2%) and up to the start of the goal-setting section (43/223, 19.3%). The introduction page to the PRIMIT study saw the highest level of attrition (304/9155, 3.3%), meaning that 53.5% (304/568) of the total attrition occurred on the first page. Completion of the core section of Germ Defence by users who had identified themselves as HCPs was 79.1% (53/67), which was similar to that of all baseline responders (169/223, 75.8%). After completing the core section, 38.8% (242/624) of all Germ Defence users accessed one or more of the 3 further components (see Figure 1) compared to 47.5% (106/223) of the Germ Defence baseline respondents.

#### Handwashing Behaviors

**Figure 5:**
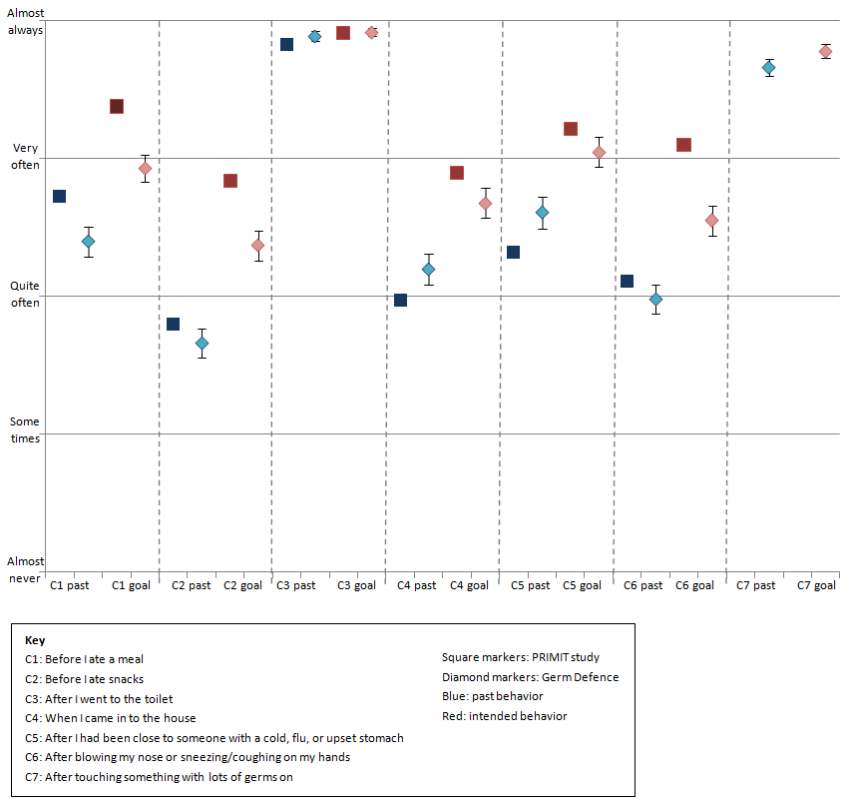
Means and confidence intervals for goal-setting behavior change technique for PRIMIT (PRImary care trial of a website based infection control intervention to Modify Influenza-like illness and respiratory infection Transmission) study and Germ Defence.

Germ Defence baseline responders and PRIMIT study users both chose to increase their intended handwashing compared to their current levels for 5 of the 7 situations (see Figure 5 and Tables S2-S4 in Multimedia Appendix 5 for mean differences and confidence intervals). Intentions to wash hands after going to the toilet did not increase in either context (Table S2 in Multimedia Appendix 5). Germ Defence users also selected similar current and intended levels for “after touching something with germs on.” Scores for HCPs using Germ Defence were not different from those of non-HCPs for both current and intended behavior (Table S4 in Multimedia Appendix 5). Owing to the large sample size, mean ranges for the PRIMIT study are smaller than those for the Germ Defence sample.

When comparing current behavior between the 2 studies, the score for “before I ate a meal” was the only practically significant situation (mean difference 0.320, 95% CI 0.121-0.518), with PRIMIT study users washing their hands more frequently (mean 3.73) than Germ Defence users (mean 3.41) (Table S3 in Multimedia Appendix 5). Scores for intended behavior showed that PRIMIT study users had selected higher frequencies (mean 4.10) for “after blowing my nose, sneezing/coughing on my hands” (mean difference 0.381, 95% CI 0.206-0.557) compared to Germ Defence users (mean 3.72).

## Discussion

### Overview of This Study

This paper presents process evaluations of an intervention applied in 2 different contexts to lower the transmission of viruses at home by increasing handwashing. The intervention was used in an RCT by National Health Service patients recruited via their general practices (PRIMIT study) and as an open access intervention available to the general public (Germ Defence). By comparing the 2 contexts, we hope to better understand any effect that the design of the RCT had on who accessed the intervention, how the intervention was used, and whether the intervention continued to increase handwashing intentions once disseminated to the public.

### Implications for Web-Based Research Enrollment Procedures

Half the PRIMIT RCT participants dropped out during the web-based research enrollment process prior to reaching the intervention. This intervention had been developed in line with the person-based approach; accessibility and engagement with content and design were optimized through think-aloud interviews and survey responses [3]. However, although baseline measures were assessed quantitatively to ensure they were valid [17], additional patient and public involvement or qualitative assessment during development would have provided information on the impact and acceptability of these research processes. This would have allowed the opportunity to improve them and reduce the risk of losing a substantial number of potential users before they have even reached the intervention. The Germ Defence study lost a third of the participants during research enrollment. Considerable effort was invested to develop and optimize the consent and baseline measures for Germ Defence. The process was made easier and quicker than that for PRIMIT with optional rather than compulsory baseline measures. These pages were constructed in line with the design values and language used for the original intervention [3,17] and were additionally tested for usability by 8 researchers experienced in person-based digital intervention development. As a preliminary dissemination study, these procedures were necessary to compare participant characteristics and handwashing behaviors of the Germ Defence study to those from the PRIMIT study. As with the PRIMIT RCT, the potential impact of this on uptake had not been assessed in advance. However, by making the process voluntary, we were able to demonstrate that over 60% of Germ Defence users preferred not to complete baseline measures at all. Almost 80% of the first visits to the Germ Defence website did not progress past the first page. The data available do not offer explanation for the high initial dropout. One possible explanation could be that the intervention was first developed in 2008 and intended to be used on personal computers and larger screen tablets; the website was unable to responsively adapt to mobile viewing (eg, users needed to enlarge or scroll across the page if viewing content on a mobile phone screen). This may have discouraged a considerable number of users. In addition, the number of visits at this point may include multiple visits by the same people; consequently, users who visited the first page and then decided to return at a later point to view the whole intervention cannot be identified within the attrition rate.

### Implications for Intervention Usage

High levels of attrition during research enrollment procedures are a concern as they may lead to selection bias in the remaining sample. Comparison of intervention usage between the 2 contexts highlighted this effect: over 90% of PRIMIT study users completed the core section of the intervention compared to 65% of all Germ Defence users. This suggests that effortful enrollment research procedures and high levels of dropout may have resulted in a remaining sample that was more motivated to use the intervention. This is supported within Germ Defence where the completion rate increased to 75% for users who had participated in baseline measures, and they were more likely to view the additional components within the intervention. As none of the user characteristics recorded at baseline (eg, handwashing behavior, perceived likelihood of becoming ill) were associated with completing the core section of the intervention, explanations for this increased usage may be explained by alternative characteristics (eg, motivation to learn about infection prevention behaviors, altruism). As suggested above, extending qualitative methods used to develop intervention content to include research procedures may also help identify characteristics associated with engagement with the research as well as with target behavior.

The majority of attrition from the intervention occurred across the first few pages in both studies. Germ Defence users may have chosen to leave as they were dissatisfied with the content, or alternatively, given that these pages received positive feedback during development, users may have felt they had found sufficient information and did not need to continue. Further research is required to understand this attrition, particularly for the introduction page of the PRIMIT study. This page contained no “active ingredients,” yet saw the highest proportion of users leave despite having completed the effortful enrolment process. The page with the highest proportion of attrition in Germ Defence was the first page of the goal-setting section. This is the first time users were required to interact with the intervention, and they were unable to progress to the next page without entering current handwashing frequencies. Although users may have decided to leave rather than complete an activity they considered to be too effortful, the goal-setting section was seen to be effective at raising intentions to wash hands more in the future.

### Implications for Promoting Increased Handwashing

When comparing PRIMIT study and Germ Defence handwashing selections for the goal-setting BCT, users in both contexts intended to improve their frequency of handwashing in the future in 5 situations (ie, before meals, before snacks, after coming in to the home, after being close to someone who is ill, after sneezing or coughing). Minimal improvement was seen for washing hands after going to the toilet and touching something with germs on. This is probably due to the high levels already reported for current behavior. Interestingly, users reported only washing their hands “quite often” on average after blowing their nose, sneezing, or coughing, despite repeated public health campaigns targeting this specific behavior in the United Kingdom [38]. Qualitative research during intervention development highlighted that people found this situation difficult to carry out due to lack of control [2,3]. However, users did chose to improve on this behavior in the future having viewed the intervention.

The goal-setting section helped Germ Defence users to plan improvements in handwashing in the same way as had been seen in the PRIMIT study RCT for 5 of the 6 shared situations. This suggests that the intervention mechanisms work as effectively when disseminated to the general public as they did in the RCT and that the reduction of illnesses seen in PRIMIT study sample is likely to be replicated in the Germ Defence sample. These similar levels of intended handwashing occurred despite the RCT experiencing greater levels of selection bias as discussed above, leading to a more motivated sample. An explanation for this may be found in the Germ Defence sample where almost a quarter of baseline responders were HCPs. At baseline, HCPs indicated higher amounts of both current and intended daily handwashing than non-HCPS. Yet, when completing the goal-setting section, HCPs and non-HCPs showed similar levels of current and increased intended future handwashing for the specific situations within the BCT. This suggests that although HCPs wash their hands more frequently, they might be overlooking handwashing opportunities in the home that are important for reducing infection transmission. If this effect was seen with HCPs using Germ Defence, then it is likely that using these specific situations was also effective for highly motivated users in the PRIMIT study.

### Application of the Findings to Disseminate Germ Defence for Use in a Pandemic

In March 2020, Germ Defence was adapted and disseminated globally for use in the COVID-19 pandemic [31,32]. The findings from this preliminary dissemination study were applied to the intervention to maximize uptake and reach. Having established that even minimal research enrolment procedures may act as a barrier to usage, all baseline measures were removed for the Germ Defence COVID-19 intervention so that users could access the intervention as quickly and easily as possible [31]. Instead, after completing the core section, users are invited to take part in a survey where some demographic information is collected along with questions about using the intervention. While the sample that reaches this stage of the intervention will be biased, the survey has still collected valuable and insightful information [39]. In addition, the goal-setting section embedded within the intervention provides a measure of past/intended behavior. To reduce attrition within the goal-setting section, an additional message explaining the value of completing the measures has been added, which is hoped will encourage participation.

### Limitations

Comparing the large sample in the PRIMIT study to the much smaller number of users who accessed Germ Defence is problematic because they are likely to differ in many respects, which could not be evaluated. However, comparisons are often made between implementation of interventions in different contexts, and given the difference in contexts and probably also populations, it is notable that beliefs at baseline and self-reported behaviors were similar in the trial and the implementation. The difference in the number of participants is likely to reflect recruitment methods: the PRIMIT study sent letters directly to over 800,000 people, whereas Germ Defence relied heavily on health organizations to distribute the details. The low level of Germ Defence users suggests that prevention of seasonal colds and flu may not be a strong motivation for many people (Germ Defence for COVID-19 has seen considerably higher usage) [30]. This means that people who did use Germ Defence may have been more highly motivated to practice good infection prevention behaviors, and given the low proportion of uptake for PRIMIT, the same may be said of that sample. Yet, despite this, the intervention group for the PRIMIT study experienced lesser respiratory tract infections, and selections for the goal-setting section demonstrated the ability of the intervention to help users in both studies to increase their handwashing.

The large proportion of dropouts seen on the first page of Germ Defence is unexplained. This is a common phenomenon of intervention usage in the community [40], and as such, including qualitative data collection in future process evaluations would provide some insight for this problem and inform further refinement of the first page to maximize usage.

### Conclusions

This study provides an example of how interventions assessed through RCTs can be examined and adapted to try to optimize their usage when disseminated to the general public. By comparing RCT data to a novel preliminary dissemination study, we were able to examine our 3 aims. First, this research demonstrates that the Germ Defence intervention continues to raise users’ intentions to wash their hands more in specific situations where the risk of virus transmission is high, replicating the behavior changes reported during the PRIMIT RCT. The responses show that using a goal-setting BCT was helpful for increasing intended handwashing, including in situations identified as being particularly difficult, despite having been already targeted by long-term public health messages. Second, we established that the effortful web-based enrolment procedures required for the PRIMIT RCT led to participant dropout, acting as a barrier to accessing health information. Third, the effect of this was apparent through our aim of comparing intervention usage, as the remaining PRIMIT users showed higher levels of usage than those accessing the intervention in the Germ Defence study. By designing the Germ Defence study to observe and compare the preliminary dissemination of the intervention, we were able to establish points of increased attrition within the intervention. The findings provided the opportunity to adapt and improve Germ Defence for wider dissemination and during the onset of COVID-19 for a public health emergency.

## Appendix

Multimedia Appendix 1 Stage 1 checklist of AMUsED (Analyzing and Measuring Usage and Engagement Data) framework: Familiarization with the data. Completed for the PRIMIT (PRImary care trial of a website based infection control intervention to Modify Influenza-like illness and respiratory infection Transmission) study and Germ Defence.

Multimedia Appendix 2 Stage 2 checklist: selecting usage variables and generating research questions.

Multimedia Appendix 3 Stage 3 checklist: preparation for analysis.

Multimedia Appendix 4 Results of the logistic regression analysis of baseline user characteristics predicting the completion of core sections of PRIMIT (PRImary care trial of a website based infection control intervention to Modify Influenza-like illness and respiratory infection Transmission) and Germ Defence intervention.

Multimedia Appendix 5 Mean differences and 95% confidence intervals for goal-setting section on handwashing frequencies.

## Abbreviations

AMUsED: Analyzing and Measuring Usage and Engagement Data
BCT: behavior change technique
HCP: health care professional
NIHR: National Institute for Health Research
PRIMIT: PRImary care trial of a website based infection control intervention to Modify Influenza-like illness and respiratory infection Transmission
RCT: randomized controlled trial
