# Supplementary material for: A Web-Based Intervention (Germ Defence) to Increase Handwashing During a Pandemic: Process Evaluations of a Randomized Controlled Trial and Public Dissemination
Source: J Med Internet Res. 2021 Oct 5;23(10):e26104. doi: 10.2196/26104 (PMC8494071; doi:10.2196/26104)
Supplement: Multimedia Appendix 1 [file jmir_v23i10e26104_app1.docx]

**Multimedia Appendix 1.** Stage 1 checklist of AMUsED (Analyzing and Measuring Usage and Engagement Data) framework: Familiarization with the data. Completed for the PRIMIT (PRImary care trial of a website based infection control intervention to Modify Influenza-like illness and respiratory infection Transmission) study and Germ Defence.

| Familiarisation with the data – identifying variables | | | |
| --- | --- | --- | --- |
| Generic questions by data type | Intervention: *PRIMIT* | | Intervention: *Germ Defence* |
| **1. Intervention characteristics. Data for intervention architecture and content.** | | | |
| **1.1 Workflow. Intervention structure and expected participant interaction and navigation through the intervention.** | | | |
| How many logins/sessions are available? | *4 sessions.* | *Change to 1 session (see previous findings 3.2) with ‘core’ section based on 1^st^ PRIMIT session.* | |
| When are they available? | *1^st^ at baseline, 2^nd^ released 3.5 days post-baseline, 3^rd^ at 10.5 days, 4^th^ at 17.5 days post-baseline.* | *Intervention available at once* | |
| Are new sessions released depending on time elapsed or task-completion? | *Released after the times set, and dependent on having accessed previous session.* | *n/a* | |
| Are there limitations on the availability of the intervention? | *Previous session must be accessed in order to view next. Content from previous sessions could be revisited in subsequent session.* | *n/a* | |
| Is the purpose of a session to collect self-report measures and/or use the intervention? | *Session 1 includes baseline measures and content, all other sessions are content alone.* | *Follow-up survey contained no intervention content* | |
| When is the intervention considered to be finished? | *At the end of the 4^th^ session.* | *After completing the core section* | |
| What prompts are used to encourage usage (e.g. emails, texts, notifications) and when are they sent? | *An email is sent when next session is ready, followed by 2 further emails if they don’t login.* |  | |
| Does the intervention contain ‘tunneled’ (compulsory) sequences of pages which users have to view to move forward? | *Initial pages of each session are tunneled.* | *Core section is tunneled* | |
| Are users able to select linked components they wish to view, and avoid others? | *Optional menu components are available after completing tunneled pages.* | *After completing the core section, 3 menu components will be available using content from sessions 2-4.* | |
| **1.2. Content. Content available within the pages of the intervention.** | | | |
| What are the components available? | *Tunneled components in sessions 1-4 providing information on handwashing. Menu components: more advice on looking after someone with flu, details about the research, revisiting goal-setting, revisiting information from previous sessions.* | *Core component from session 1. Menu components for more advice on handwashing, flu, and details about the research. There is also the opportunity to return to the start of the core section again.* | |
| What is the aim of each component and are they based on underlying theoretical constructs? | *Messages based on the theory of planned behaviour* [38] *promote handwashing as an effective behaviour (positive attitudes), socially desirable (subjective norms), and easy to do (perceived behavioral control). Messages utilizing protection motivation theory* [39] *provided information on health consequences and infection transmission for RTIs.* | *Same as PRIMIT* | |
| In what order is it anticipated the components will be used? | *Tunneled components have to be used first. No expectation for menu components.* | *Core section first* | |
| What interactive features are available (e.g. forums, videos, printable information)? How long should they take to complete? | *Goal setting component - users with low intended handwashing who select no intention to improve receive supportive messages encouraging them to review their choices. An optional print-out page is available.* | *Same as PRIMIT* | |
| Are all components/features available to all users throughout the intervention or are some tailored for specific times or users? | *Users self-report amount of handwashing, efficacy and necessity beliefs at the start of sessions 2-4. Users with low handwashing and beliefs are presented with additional tailored information during the tunneled component. Some content is also tailored depending on household type (e.g. for users with children).* | *No tailoring* | |
| Which pages are for collecting self-report measures or for administrative purposes | *Page names starting with ‘s’ or ‘t’ indicate active content.* | *4 intro pages before the intervention to collect voluntary baseline measures* | |
| Are there specific pages to mark the start and end of sessions? | *First page of tunneled component marks start of each session.* | *n/a* | |
| Which pages contain BCTs (e.g. information, planning, feedback) and what are they? | *Tunneled pages at the start of each session contain: messages to increase perceived risk, information for explanation of infection transmission by hand, motivation to increase positive attitudes to handwashing, information on viral load and washing hands to reduce infection, goal setting. Tailored pages within tunneled components contain messages to support: habit formation, overcoming barriers, understanding necessity.* | *Tunneled pages (core section) as for PRIMIT* | |
| In which sessions are they available? | *Across all 4.* | *1 session* | |
| Can specific BCTs be identified on particular pages or groups of pages? How many groups are there? | *Goal setting in session 1 provides cues and feedback over 4 pages. Tailored pages across sessions 2-4 are grouped by handwashing, necessity, and efficacy. Pages addressing attitudes, barriers and beliefs are used throughout the tailored pages.* | *As PRIMIT, but without tailored pages* | |
| Do any of the pages have response options to collect information in addition to baseline/follow-up measures? What data is collected? | *Goal setting pages collect actual and intended handwashing plan. However, repeat use within a session is not recorded, only the last entry is stored.* | *Goal setting pages: amended Germ Defence to capture repeated page viewings and goal entries up to 3 times.* | |
| **2. Accrued data. Data collected during an intervention.** | | | |
| **2.1. Self-report. Users’ self-reported responses collected across various stages of the trial.** | | | |
| When are self-report questionnaires collected? | *Baseline and every 4 weeks up to 12 weeks.* | *Optional baseline and follow-up survey completed one week later.* | |
| What demographic information is available (e.g. age, gender, education)? | *Baseline: Age, gender, qualification, household.* | *Same as PRIMIT , plus item for how they heard about the intervention.* | |
| Which measures are specifically related to the target behaviour and how often are they collected? | *Baseline, session 2-4, 4 & 12 weeks: Actual and intended handwashing behaviour. Every 4 weeks: User and household illness occurrence.* | *For dissemination: follow-up measures at 1 week for current and intended handwashing behaviour, necessity and efficacy.* | |
| Which measures of target behavioral determinants are collected and when? | *Baseline, 4 & 12 weeks: attitudes, norms, PBC, current & intended handwashing. Perceived risk: likelihood of catching.* | *Baseline & 1 week: attitudes, norms, PBC, current & intended handwashing. Perceived risk: likelihood of catching& severity* | |
| Are measures of health collected (e.g. conditions which may impact on target behaviour or are co-morbid) and psychosocial factors (e.g. anxiety, illness perception, motivation)? | *Flu vaccination.* | *No* | |
| Are additional measures collected at follow-up (e.g. satisfaction, adherence)? | *Week 8: Satisfaction questions.* | *Acceptability e-scale* [40]^.^ | |
| **2.2. Log-data. Information automatically collected through engagement with an intervention.** | | | |
| What data is the software platform able to record? | *Time and date, pages viewed & order, time spent on pages, self-report measures.* | *Same as PRIMIT* | |
| Are number, date and time of logins available by individual user? | *Yes.* | *Same as PRIMIT* | |
| Are individuals’ total durations of usage accessible? | *Needs to be extracted.* | *Same as PRIMIT* | |
| Are the number and time of usage prompts recorded? | *Overall scheduled timings for emails are available, but not sent times by individual.* | *Same as PRIMIT* | |
| Are there details for which pages were viewed, the sequential order and time spent viewing? | *Yes.* | *Same as PRIMIT* | |
| **2.3. External data. Data collected independently but alongside intervention usage** | | | |
| How and where is the data collected? | *Hand collected by the research team from users’ GP notes.* | *n/a* | |
| What data is collected? | *Visits to a GP for an RTI or gastrointestinal infection during trial period. Antibiotic prescriptions for RTI.* |  | |
| Which of these measures relate to or may impact on the target behaviour? | *Number of GP visits for RTI by user* |  | |
| **3. Contextual data. Data indirectly related to the running of the intervention which may be influential over usage and analysis.** | | | |
| **3.1. External factors. Structures and events which may influence participation in the intervention.** | | | |
| How are users recruited to the intervention? | *Recruited via GP using paper consent.* | *Website promoted to health support groups, via NICE and direct to community. Changed to online consent* | |
| Did any specific large-scale events, with the potential to impact on the intervention, occur during the period of the intervention? | *Outbreak of swine flu prior to trial commencing.* | *None* | |
| **3.2. Previous theory and findings. Results of behavioral analyses carried out during intervention development (e.g. logic models), and analyses of clinical outcomes if available.** | | | |
| What are the hypothesized mechanisms of the intervention (e.g. as specified in the intervention’s logic model)? | *Handwashing will increase when intentions to wash hands are raised through positive attitudes, norms and beliefs. Increasing perceptions of risk from an RTI will lead to increased motivation to handwash.* | *Same as PRIMIT* | |
| Which factors are identified as important in qualitative research, and can they be related to the variables collected in the trial? | *N/a* |  | |
| Which variables are identified as relating to outcomes (e.g. behavioral determinants, theoretical constructs, health factors)? | *Perceived risk of infection was a key predictor of intentions to handwash* [18]*.* | *Baseline measures include perceived risk* | |
