# Supplementary material for: A Web-Based Intervention (Germ Defence) to Increase Handwashing During a Pandemic: Process Evaluations of a Randomized Controlled Trial and Public Dissemination
Source: J Med Internet Res. 2021 Oct 5;23(10):e26104. doi: 10.2196/26104 (PMC8494071; doi:10.2196/26104)
Supplement: Multimedia Appendix 2 [file jmir_v23i10e26104_app2.docx]

**Multimedia Appendix 2.** Stage 2 checklist: selecting usage variables and generating research questions.

| Selecting usage variables and generating research questions | | |
| --- | --- | --- |
| Generic questions | Intervention: *PRIMIT* | Intervention: *Germ Defence* |
| 1. **Descriptions of usage variables. Which usage variables are relevant to the intervention** **and in which format (e.g. number of users/sessions, duration, percentage of total, dichotomous)?** | | |
| Completing intervention/trial period (stage 1; 1.1 & 2.2). | *Number of users completing session 1. Number of pages viewed (including baseline measures and tunneled). Time spent by page.* | *Number of users completing core section. Number of pages viewed (including baseline measures, tunneled and individual menu components). Time spent by page.* |
| Logins or sessions where the intervention was accessed (stage 1; 1.1 & 2.2). | *Session 1 only* | *N/a* |
| Date of login and usage. | *N/a* |  |
| Time of day of login and usage. | *N/a* |  |
| Days/weeks of usage (stage 1; 1.1 & 2.2). | *N/a* |  |
| Response to prompts/notifications (stage 1; 1.1 & 2.2). |  | *Number of users accessing the survey.*  *Number of users completing the survey.* |
| Features/menu components used (stage 1; 1.1, 1.2 & 2.2). | *Number of users viewing printout page.* | *Number of users viewing each menu component. Number of pages viewed in menu components. Number of users viewing printout page.* |
| Revisiting components/features (stage 1; 1.1, 1.2 & 2.2). | *Number of users receiving tailored feedback for low or no improvement in behavior who revisit goal setting component.* | *Number of users receiving tailored feedback for low or no improvement in behavior who revisit goal setting component. Number of users returning to the start of the tunneled component from the menu page.* |
| Type of content/BCTs used (excluding administration pages) (stage 1; 1.2 & 2.2). | *User dropout by page (including consent and baseline measures pages).* | *Same as PRIMIT* |
| Completing ongoing measures (stage 1; 2.1 & 2.2). |  | *Number of users entering their email address to take part in the survey. Number of questions completed in survey. Number of users completing each question.* |
| External device usage (stage 1; 2.3). | *N/a* | *n/a* |
| **2. Relationships between usage and participant characteristics. Are user’s demographic, physical or psychosocial characteristics at baseline related to intervention usage?** | | |
| Are any characteristics at baseline related to usage? | *Is age, gender or education related to usage? Is type of household related to usage? Are perceived likelihood and/or severity for user or a member of their household related to usage* [39]*?* | Same as PRIMIT |
| Are any contextual factors related to usage (stage 1; 3)? |  | *Does how users hear about the website relate to usage?* |
| Do high/low users differ by other usage factors? |  | *Is there a relationship between intervention usage and survey usage?* |
| **3. Relationships between usage, behavioral determinants, and target behaviors. Which usage variables are associated with follow-up measures for target behaviour and behavioral determinants? Which usage variables help explain changes in behaviour across the intervention?** | | |
| Are baseline measures for behavioral determinants/target behavior related to usage? E.g. Is the number of days the intervention is used for related to a behavioral determinant? Do users with low target behavior spend less time on the intervention? | *Is handwashing level at baseline related to usage? Is perceived necessity or efficacy of handwashing related to usage?* | Same as PRIMIT |
| Which usage variables are related to behavioral determinants/target behaviors and at follow-up? E.g. Do users who view a group of pages containing a specific BCT score higher/lower for the associated behavioral determinant? Is completing/not completing a particular component associated with target behavior at follow-up? Is the time spent on a session related to target behavior? |  | *Which usage variables are related to handwashing in the survey? Which usage variables are related to perceived necessity, and efficacy?* |
| Is usage associated with measures for acceptability/satisfaction at follow-up? E.g. Are high levels of satisfaction associated with accessing more pages? Do users with low satisfaction spend less time using external devices? |  | *Which usage variables are associated with scores for user satisfaction, and acceptability e-scale measures* [40]*?* |
| Do users who report positive changes in behavioral determinants/target behavior from baseline to follow-up use the intervention differently to those who do not? | *Do users who report increases in handwashing use the website differently to those who don’t report increases? Do users who report increases in perceived necessity and efficacy use the intervention differently?* | *Same as PRIMIT* |
| Are relationships between usage and outcome measures moderated by demographic, psychosocial or health factors? | *Does perceived level of risk* [39] *moderate the relationship between usage and increased handwashing?* | *Does perceived level of risk* [39] *moderate the relationship between usage and increased handwashing?* |
| What level of usage is necessary for ‘effective engagement’? | *Do any of the usage variables have a point of sufficient engagement where maximum behaviour change has occurred?* | *Do any of the usage variables have a point of sufficient engagement where maximum behaviour change has occurred?* |
