# Supplementary material for: A Web-Based Intervention (Germ Defence) to Increase Handwashing During a Pandemic: Process Evaluations of a Randomized Controlled Trial and Public Dissemination
Source: J Med Internet Res. 2021 Oct 5;23(10):e26104. doi: 10.2196/26104 (PMC8494071; doi:10.2196/26104)
Supplement: Multimedia Appendix 3 [file jmir_v23i10e26104_app3.docx]

Table 3. AMUsED Framework Stage 3 Checklist for PRIMIT Study and Germ Defence

| Preparation for analysis | | | | | | | | | |
| --- | --- | --- | --- | --- | --- | --- | --- | --- | --- |
| Generic questions | Intervention: *PRIMIT* | | | | | Intervention: *Germ Defence* | | | |
| **1. Resources** | | | |  | | | |  | |
| What is the timeframe for completing the analyses? | | | *Start of 2020.* | | | | *Same as PRIMIT* | |  |
| What resources are needed? E.g. additional research time, expertise | | |  | | | | *At least 200 users.*  *Support with selection of analyses.* | |  |
| Is a plan of analysis already available? How does the analysis plan developed using the framework compare to that plan? Are changes or updates needed? | | | *No other plan available.* | | | |  | |  |
| Is ethical clearance in place to carry out usage analyses? | | | *Yes.* | | | | *Yes* | |  |
| **2. Selecting types of analysis and analytical software** | | |  | | | |  | |  |
| Will the usage data be triangulated with qualitative data? | | | *No.* | | | | *No* | |  |
| What analytical tools are available? | | | *SPSS & LifeGuide Visualisation Tool (LVT).* | | | |  | |  |
| Is there sufficient statistical power to answer the planned research questions? | | |  | | | | *No, analyses will be exploratory.* | |  |
| Can the selected measures of usage be analyzed using the available tools? Is bespoke software necessary (e.g. visualisation techniques)? | | | *All measures can be processed using SPSS* | | | |  | |  |
| **3. Data preparation** | | |  | | | |  | |  |
| When is the data available? | | | *From spring 2019.* | | | | *Summer 2019 onwards* | |  |
| Is the data raw or has it been used/cleaned previously? | | | *Raw log-data, self-report measures on cleaned SPSS file* | | | | *Raw log-data.* | |  |
| How many datasheets are there? Will these need to be amalgamated? | | | *3 raw datasets (1 from each winter) containing 4 sheets each with log data. 1 SPSS sheet used previously for RCT behavioral analysis.*  *These will need to be amalgamated to make 1 raw dataset with 4 sheets, then user IDs matched to RCT users on SPSS.* | | | | *8 datasheets: 4 log-data sheets for both intervention and survey. Survey data will be matched to intervention data by assigned ID.* | |  |
| Is the data structured to work with the tools available? What formats are the datasheets in (e.g. excel, .csv) and will they need converting for analysis? | | *Datasheets are compatible with SPSS and LVT. Excel sheets will need converting to .csv for LVT, and uploaded to SPSS.* | | | *Same as PRIMIT* | | | | |
| What preparation does the data need (e.g. cleaning, anonymizing)? | | *Data needs cleaning.* | | | *Same as PRIMIT* | | | | |
| Are all variables readily available or will they need extracting/transforming/recoding? | | *Identified variables will be available. However, totals for time and numbers of pages viewed will need extracting.* | | | *Same as PRIMIT* | | | | |
| Is the data in the right format to answer the research questions? Will it need adapting (e.g. continuous variables changed to categorical)? | | *Yes* | | | *Yes* | | | | |
