# Supplementary material for: A Web-Based Intervention (Germ Defence) to Increase Handwashing During a Pandemic: Process Evaluations of a Randomized Controlled Trial and Public Dissemination
Source: J Med Internet Res. 2021 Oct 5;23(10):e26104. doi: 10.2196/26104 (PMC8494071; doi:10.2196/26104)
Supplement: Multimedia Appendix 4 [file jmir_v23i10e26104_app4.docx]

Multimedia Appendix 4. Table S1. Results for logistic regression analysis of baseline user characteristics predicting completion of core section of PRIMIT and Germ Defence intervention

| **Predictors** | | ***β*** | ***SE*** | **Wald** | ***df*** | ***P*** | **Exp(B)** | **95% CI** | |
| --- | --- | --- | --- | --- | --- | --- | --- | --- | --- |
|  | |  |  |  |  |  |  | **Lower** | **Upper** |
|  |  |  |  |  |  |  |  |  |  |
| **Gender** | |  |  |  |  |  |  |  |  |
|  | PRIMIT Study | -0.079 | 0.083 | 0.840 | 1 | 0.359 | 0.927 | 0.788 | 1.090 |
|  | Germ Defence | 0.445 | 0.362 | 1.510 | 1 | 0.219 | 1.561 | 0.767 | 3.175 |
| **Age** | |  |  |  |  |  |  |  |  |
|  | PRIMIT Study | 0.000 | 0.003 | 0.005 | 1 | 0.942 | 1.000 | 0.994 | 1.006 |
|  | Germ Defence | 0.009 | 0.013 | 0.458 | 1 | 0.449 | 1.009 | 0.983 | 1.035 |
| **Current daily handwashing** | |  |  |  |  |  |  |  |  |
|  | PRIMIT Study | 0.111 | 0.072 | 2.386 | 1 | 0.122 | 1.117 | 0.971 | 1.286 |
|  | Germ Defence | 0.228 | 0.225 | 1.029 | 1 | 0.310 | 1.256 | 0.808 | 1.953 |
| **Intended daily handwashing** | |  |  |  |  |  |  |  |  |
|  | PRIMIT Study | -0.068 | 0.075 | 0.823 | 1 | 0.364 | 0.934 | 0.806 | 1.082 |
|  | Germ Defence | -0.002 | 0.229 | 0.000 | 1 | 0.994 | 0.998 | 0.638 | 1.563 |
| **Perceived likelihood of user becoming ill** | |  |  |  |  |  |  |  |  |
|  | PRIMIT Study | 0.006 | 0.024 | 0.060 | 1 | 0.806 | 1.006 | 0.959 | 1.055 |
|  | Germ Defence | 0.214 | 0.115 | 3.431 | 1 | 0.064 | 1.238 | 0.988 | 1.553 |
| **Perceived severity for user** | |  |  |  |  |  |  |  |  |
|  | PRIMIT Study | - | - | - | - | - | - | - | - |
|  | Germ Defence | -0.131 | 0.107 | 1.511 | 1 | 0.219 | 0.877 | 0.711 | 1.081 |
| *SE* = standard error, *df* = degrees of freedom, *p* = significance, OR = odds ratio, CI = confidence interval | | | | | | | | | |
