# Supplementary material for: A Web-Based Intervention (Germ Defence) to Increase Handwashing During a Pandemic: Process Evaluations of a Randomized Controlled Trial and Public Dissemination
Source: J Med Internet Res. 2021 Oct 5;23(10):e26104. doi: 10.2196/26104 (PMC8494071; doi:10.2196/26104)
Supplement: Multimedia Appendix 5 [file jmir_v23i10e26104_app5.docx]

Multimedia appendix 5. Tables S2-S4. Mean differences and 95% confidence intervals for goal-setting section handwashing frequencies

**Table S2.** Comparison of mean scores for current vs intended levels of handwashing within the same study/intervention

| Situation | PRIMIT study | | | Germ Defence | | |
| --- | --- | --- | --- | --- | --- | --- |
|  | *MD* | 95% CI | | *MD* | 95% CI | |
|  |  | Lower | Upper |  | Lower | Upper |
| C1 (before eating meals) | *0.654* | *0.634* | *0.673* | *0.718* | *0.557* | *0.878* |
| C2 (before eating snacks) | *1.040* | *1.016* | *1.063* | *0.949* | *0.766* | *1.132* |
| C3 (after going to the toilet) | 0.079 | 0.072 | 0.086 | 0.040 | -0.003 | 0.082 |
| C4 (coming in to the house) | *0.930* | *0.907* | *0.953* | *0.661* | *0.502* | *0.820* |
| C5 (after being close to someone who is ill) | *0.902* | *0.878* | *0.926* | *0.768* | *0.575* | *0.961* |
| C6 (after sneezing or coughing) | *0.995* | *0.972* | *1.019* | *0.842* | *0.675* | *1.008* |
| C7 (after touching something with germs on) | - | - | - | 0.092 | 0.192 | 0.292 |

**Table S3**. Comparison of mean scores between PRIMIT vs Germ Defence studies for current behaviour and intended behaviour

| Situation | Current | | | Intended | | |
| --- | --- | --- | --- | --- | --- | --- |
|  | *MD* | 95% CI | | *MD* | 95% CI | |
|  |  | Lower | Upper |  | Lower | Upper |
| C1 (before eating meals) | *0.305* | *0.108* | *0.502* | 0.261 | 0.112 | 0.409 |
| C2 (before eating snacks) | 0.146 | -0.062 | 0.355 | 0.237 | 0.047 | 0.428 |
| C3 (after going to the toilet) | -0.063 | -0.148 | 0.022 | -0.021 | -0.084 | 0.041 |
| C4 (coming in to the house) | -0.229 | -0.449 | -0.009 | 0.041 | -0.149 | 0.231 |
| C5 (after being close to someone who is ill) | 0.084 | -0.140 | 0.308 | 0.207 | 0.035 | 0.380 |
| C6 (after sneezing or coughing) | 0.242 | 0.320 | 0.453 | *0.381* | *0.206* | *0.557* |
| C7 (after touching something with germs on) | - | - | - | - | - | - |

Table S4. Comparison of mean scores between HCPs vs non-HCPs for Germ Defence

| Situation | Current | | | Intended | | |
| --- | --- | --- | --- | --- | --- | --- |
|  | *MD* | 95% CI | | *MD* | 95% CI | |
|  |  | Lower | Upper |  | Lower | Upper |
| C1 (before eating meals) | 0.044 | -0.422 | 0.510 | -0.096 | -0.479 | 0.288 |
| C2 (before eating snacks) | 0.117 | -0.336 | 0.570 | 0.204 | -0.247 | 0.655 |
| C3 (after going to the toilet) | -0.109 | -0.258 | 0.039 | -0.052 | -0.161 | 0.056 |
| C4 (coming in to the house) | -0.115 | -0.607 | 0.377 | -0.203 | -0.633 | 0.227 |
| C5 (after being close to someone who is ill) | 0.216 | -0.355 | 0.788 | 0.150 | -0.321 | 0.620 |
| C6 (after sneezing or coughing) | 0.149 | -0.319 | 0.617 | 0.055 | -0.403 | 0.512 |
| C7 (after touching something with germs on) | 0.052 | -0.254 | 0.358 | 0.009 | -0.189 | 0.207 |

Practically significant results (*MD*>0.3) are in bold.
